# A multi-ethnic meta-analysis confirms the association of rs6570507 with adolescent idiopathic scoliosis

**DOI:** 10.1038/s41598-018-29011-7

**Published:** 2018-08-01

**Authors:** Ikuyo Kou, Kota Watanabe, Yohei Takahashi, Yukihide Momozawa, Anas Khanshour, Anna Grauers, Hang Zhou, Gang Liu, Yan-Hui Fan, Kazuki Takeda, Yoji Ogura, Taifeng Zhou, Yusuke Iwasaki, Michiaki Kubo, Zhihong Wu, Morio Matsumoto, Japan Scoliosis Clinical Research Group (JSCRG), Noriaki Kawakami, Koki Uno, Teppei Suzuki, Hideki Sudo, Shohei Minami, Toshiaki Kotani, Manabu Ito, Haruhisa Yanagida, Hiroshi Taneichi, Ikuho Yonezawa, Kazuhiro Chiba, Naobumi Hosogane, Nobuyuki Fujita, Mitsuru Yagi, Katsuki Kono, Eijiro Okada, Kotaro Nishida, Kenichiro Kakutani, Tsuyoshi Sakuma, Katsumi Harimaya, Takashi Kaito, Kei Watanabe, Yuki Taniguchi, Taichi Tsuji, Tsutomu Akazawa, Texas Scottish Rite Hospital for Children Clinical Group (TSRHCCG), Lori A. Karol, Karl E. Rathjen, Daniel J. Sucato, John G. Birch, Charles E. Johnston, Benjamin S. Richards, Brandon Ramo, Amy L. McIntosh, John A. Herring, Elisabet Einarsdottir, Juha Kere, Dongsheng Huang, Guixing Qiu, Yong Qiu, Carol A. Wise, You-Qiang Song, Nan Wu, Peiqiang Su, Paul Gerdhem, Shiro Ikegawa

**Affiliations:** 1 Laboratory of Bone and Joint Diseases, RIKEN Center for Integrative Medical Sciences, Tokyo, Japan; 2 Department of Orthopaedic Surgery, Keio University School of Medicine, Tokyo, Japan; 3 Laboratory for Genotyping Development, RIKEN Center for Integrative Medical Sciences, Yokohama, Japan; 4 Sarah M. and Charles E. Seay Center for Musculoskeletal Research, Texas Scottish Rite Hospital for Children, Dallas, Texas USA; 5 Department of Orthopaedics, Sundsvall and Härnösand County Hospital, Sundsvall, Sweden; 6 Department of Clinical Science, Intervention and Technology (CLINTEC) Karolinska Institutet, Stockholm, Sweden; 7 Department of Spine Surgery, The First Affiliated Hospital of Sun Yat-Sen University, Guangzhou, China; 8 Department of Orthopedic Surgery, Peking Union Medical College Hospital, Peking Union Medical College and Chinese Academy of Medical Sciences, Beijing, China; 9 Department of Biochemistry, University of Hong Kong, Hong Kong, China; 10 Department of Central Laboratory, Peking Union Medical College Hospital, Peking Union Medical College and Chinese Academy of Medical Sciences, Beijing, China; 11 Beijing Key Laboratory for Genetic Research of Skeletal Deformity, Beijing, China; 12 Medical Research Center of Orthopedics, Chinese Academy of Medical Sciences, Beijing, China; 13 Folkhälsan Institute of Genetics, and Molecular Neurology Research Program, University of Helsinki, Helsinki, Finland; 14 Department of Biosciences and Nutrition, , Karolinska Institutet, Huddinge, Sweden; 15 Department of Medical and Molecular Genetics, King’s College London, Guy’s Hospital, London, UK; 16 Department of Orthopedics, Sun Yat-Sen Memorial Hospital of Sun Yat-Sen University, Guangzhou, China; 17 Department of Spine Surgery, The Affiliated Drum Tower Hospital of Nanjing University Medical School, Nanjing, China; 18 McDermott Center for Human Growth and Development, University of Texas Southwestern Medical Center at Dallas, Dallas, Texas USA; 19 Department of Pediatrics, University of Texas Southwestern Medical Center at Dallas, Dallas, Texas USA; 20 Department of Orthopaedic Surgery, University of Texas Southwestern Medical Center at Dallas, Dallas, Texas USA; 21 Department of Orthopaedics, Karolinska University Hospital, Stockholm, Sweden; 22 Department of Orthopaedic Surgery, Meijo Hospital, Nagoya, Japan; 23 Department of Orthopaedic Surgery, National Hospital Organization, Kobe Medical Center, Kobe, Japan; 24 Department of Advanced Medicine for Spine and Spinal Cord Disorders, Hokkaido University Graduate School of Medicine, Sapporo, Japan; 25 Department of Orthopaedic Surgery, Seirei Sakura Citizen Hospital, Sakura, Japan; 26 Department of Orthopaedic Surgery, National Hospital Organization, Hokkaido Medical Center, Sapporo, Japan; 27 Department of Orthopaedic Surgery, Fukuoka Children’s Hospital, Fukuoka, Japan; 28 Department of Orthopaedic Surgery, Dokkyo Medical University School of Medicine, Mibu, Japan; 29 Department of Orthopaedic Surgery, Juntendo University School of Medicine, Tokyo, Japan; 30 Department of Orthopaedic Surgery, National Defense Medical College, Tokorozawa, Japan; 31 Department of Orthopaedic Surgery, Kono Othopaedic Clinic, Tokyo, Japan; 32 Department of Orthopaedic Surgery, Saiseikai Central Hospital, Tokyo, Japan; 33 Department of Orthopaedic Surgery, Kobe University Graduate School of Medicine, Kobe, Japan; 34 Department of Orthopaedic Surgery, Kyushu University Beppu Hospital, Beppu, Japan; 35 Department of Orthopaedic Surgery, Graduate School of Medicine, Osaka University, Suita, Japan; 36 Department of Orthopedic Surgery, Niigata University Medical and Dental Hospital, Niigata, Japan; 37 Department of Orthopedic Surgery, The University of Tokyo Hospital, Tokyo, Japan; 38 Department of Orthopaedic Surgery, Toyota Kosei Hospital, Toyota, Japan; 39 Department of Orthopaedic Surgery, St. Marianna University School of Medicine, Tokyo, Japan; 40 Department of Orthopaedic Surgery, Texas Scottish Rite Hospital for Children, Dallas, Texas USA

## Abstract

Adolescent idiopathic scoliosis (AIS) is the most common type of spinal deformity and has a significant genetic background. Genome-wide association studies (GWASs) identified several susceptibility loci associated with AIS. Among them is a locus on chromosome 6q24.1 that we identified by a GWAS in a Japanese cohort. The locus is represented by rs6570507 located within *GPR126*. To ensure the association of rs6570507 with AIS, we conducted a meta-analysis using eight cohorts from East Asia, Northern Europe and USA. The analysis included a total of 6,873 cases and 38,916 controls and yielded significant association (combined *P* = 2.95 × 10^−20^; odds ratio = 1.22), providing convincing evidence of the worldwide association between rs6570507 and AIS susceptibility. *In silico* analyses strongly suggested that *GPR126* is a susceptibility gene at this locus.

## Introduction

Adolescent idiopathic scoliosis (AIS) is defined as a lateral spinal curvature that occurs without obvious cause between age 10 and skeletal maturity. The prevalence of AIS in the adolescent population is approximately 2–3%^1^ and AIS occurs predominantly in females^2,3^. AIS has been regarded as a multifactorial disease and a number of population, family and twin studies strongly suggest the importance of genetic factors in its etiology and pathogenesis^4–7^.

Genome-wide association study (GWAS) is a powerful tool to detect genetic variants associated with common diseases. Recently, several GWASs have identified several loci associated with AIS such as chromosome 10q24.31, 6q24.1, 20p11.22, and 1p36.32 loci^8–11^. Our initial GWAS identified rs11190870, a common variant on chromosome 10q24.31 that showed significant association with AIS in a Japanese cohort^8^ and subsequent multi-ethnic meta-analysis provided convincing evidence of the association^12^. In addition, recent validation studies in different populations have also reported its significant associations^13–15^. Thus, the association of the 10q24.31 locus with AIS has been confirmed in multiple studies; however, other AIS loci have not been fully investigated.

In our previous GWAS, we identified an additional genetic variant, rs6570507 on chromosome 6q24.1, associated with AIS in a Japanese cohort^16^. The association was replicated in independent cohorts of Chinese and Caucasian in the USA^16^; however, additional studies would be necessary to confirm the association and to identify the susceptibility gene at the locus.

In this study, we performed a meta-analysis using multi-ethnic cohorts of ~46,000 subjects. The result provided convincing evidence of the association of rs6570507 with AIS susceptibility, suggesting that the chromosome 6q24.1 locus is related to the global risk of AIS. *In silico* analyses strongly suggested that *GPR126* is a susceptibility gene at this locus.

## Results

### Association of rs6570507 and AIS susceptibility

**Figure 1:**
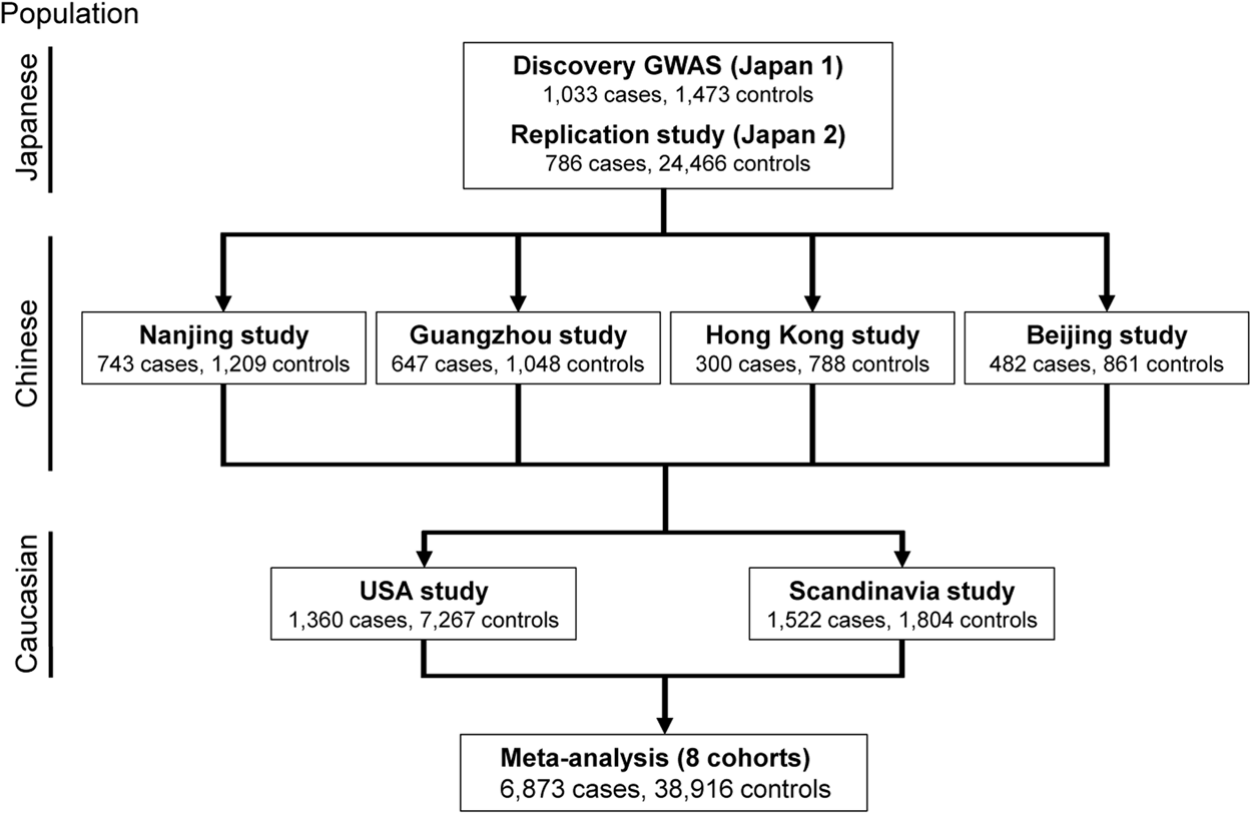
The flow diagram of the meta-analysis using eight cohorts. Three cohorts (Japanese GWAS, replication study and Nanjing study) were previously reported^16^, and the other five were recruited for this analysis.

We conducted the meta-analysis of rs6570507 using eight cohorts (Table 1, Fig. 1). Three cohorts were previously reported^16^, and the other five were recruited for this study that included cohorts from Guangzhou (case 647 and control 1,048), Hong Kong (case 300 and control 788), Beijing (case 482 and control 861), USA (case 1,360 and control 7,267), and Scandinavia (case 1,522 and control 1,804). Finally, 6,873 cases and 38,916 controls were included in the meta-analysis. The analysis showed a convincing association between rs6570507 and AIS: combined *P* = 2.95 × 10^−20^; odds ratio (OR) = 1.22; 95% confidence interval (CI) = 1.17–1.27 (Table 1, Supplementary Fig. S1). ORs were >1 in all eight cohorts, with little difference between ethnic groups. The analysis did not show any significant heterogeneity, suggesting no statistical difference between studies. Because AIS has clinical evidence of sexual dimorphism^17^, we performed gender-stratified analyses to determine whether a genetic difference existed between males and females (Table 2, Supplementary Fig. S1). However, no gender difference was observed in the association.

**Table 1:** Association of rs6570507 with adolescent idiopathic scoliosis.

Population	Study	Sample number		RAF		*P* value*	OR (95% CI)	P_het_
		Case	Control	Case	Control			
Japanese	Japan 1	1033	1473	0.49	0.42	1.37 × 10^−6^	1.32 (1.18–1.48)	
	Japan 2	786	24466	0.48	0.43	3.02 × 10^−5^	1.24 (1.12–1.37)	
	Japanese Combined	1819	25939			2.15 × 10^−10^	1.28 (1.18–1.38)	0.40
Chinese	Nanjing	743	1209	0.39	0.35	3.36 × 10^−3^	1.22 (1.06–1.39)	
	Guangzhou	647	1048	0.41	0.37	2.19 × 10^−2^	1.18 (1.02–1.36)	
	Hong Kong	300	788	0.40	0.38	4.79 × 10^−1^	1.07 (0.88–1.30)	
	Beijing	482	861	0.37	0.34	8.93 × 10^−2^	1.15 (0.98–1.36)	
	Chinese Combined	2172	3906			7.79 × 10^−5^	1.17 (1.08–1.26)	0.75
	East Asian combined	3991	29845			2.85 × 10^−13^	1.22 (1.16–1.29)	0.47
Caucasian	USA	1360	7267	0.34	0.30	7.95 × 10^−6^	1.22 (1.12–1.33)	
	Scandinavia	1522	1804	0.32	0.28	6.63 × 10^−4^	1.20 (1.08–1.34)	
	Caucasian Combined	2882	9071			1.76 × 10^−8^	1.21 (1.13–1.30)	0.85
	All combined	6873	38916			2.95 × 10^−20^	1.22 (1.17–1.27)	0.71

RAF, risk allele (rs6570507-A) frequency; OR, odds ratio; CI, confidence interval; P_het_, P-value for Cochran’s Q-test for heterogeneity.

*The p-values were calculated from the Cochran-Armitage trend test for each stage and combined p-values were calculated by the inverse variance method.

**Table 2:** Association of rs6570507 with adolescent idiopathic scoliosis by gender.

Population	Study	Female							Male						
		Sample number		RAF		*P* value*	OR (95% CI)	*P*_*het*_	Sample number		RAF		*P* value*	OR (95% CI)	*P*_*het*_
		Case	Control	Case	Control				Case	Control	Case	Control			
Japanese	Japan 1	1033	1473	0.47	0.43	1.37 × 10^−6^	1.32 (1.18–1.48)		—	—	—	—			
	Japan 2	680	9672	0.49	0.43	6.22 × 10^−5^	1.25 (1.12–1.40)		106	14794	0.47	0.43	2.27 × 10^−1^	1.18 (0.90–1.55)	
	Japanese Combined	1713	11145			3.85 × 10^−10^	1.29 (1.19–1.39)	0.50	—	—					
Chinese	Nanjing	637	711	0.39	0.35	3.69 × 10^−2^	1.18 (1.01–1.38)		104	498	0.38	0.33	1.54 × 10^−1^	1.25 (0.92–1.70)	
	Guangzhou	552	584	0.41	0.38	5.24 × 10^−2^	1.18 (0.99–1.39)		95	464	0.41	0.37	3.92 × 10^−1^	1.15 (0.84–1.58)	
	Hong Kong	248	489	0.41	0.40	7.12 × 10^−1^	1.04 (0.83–1.30)		52	299	0.37	0.36	9.36 × 10^−1^	1.02 (0.66–1.57)	
	Chinese Combined	1437	1784			8.51 × 10^−3^	1.15 (1.04–1.27)	0.62	251	1261			1.39 × 10^−1^	1.16 (0.95–1.41)	0.75
	East Asian combined	3150	12929			5.42 × 10^−11^	1.23 (1.16–1.31)	0.34	357	16055			5.62 × 10^−2^	1.17 (1.00–1.37)	0.90
Caucasian	USA	1159	4405	0.34	0.30	1.15 × 10^−4^	1.21 (1.10–1.33)		201	2862	0.34	0.29	6.65 × 10^−2^	1.22 (0.99–1.51)	
	Scandinavia	1315	1804	0.32	0.28	2.43 × 10^−3^	1.19 (1.06–1.32)		—	—	—	—			
	Caucasian Combined	2474	6209			9.12 × 10^−7^	1.20 (1.12–1.29)	0.79	—	—					
	All combined	5624	19138			2.95 × 10^−16^	1.22 (1.16–1.28)	0.56	558	18917			8.65 × 10^−3^	1.19 (1.04–1.35)	0.95

RAF, risk allele (rs6570507-A) frequency; OR, odds ratio; CI, confidence interval; P_het_, P-value for Cochran’s Q-test for heterogeneity.

*The p-values were calculated from the Cochran-Armitage trend test for each stage and combined p-values were calculated by the inverse variance method.

### Fine mapping

**Figure 2:**
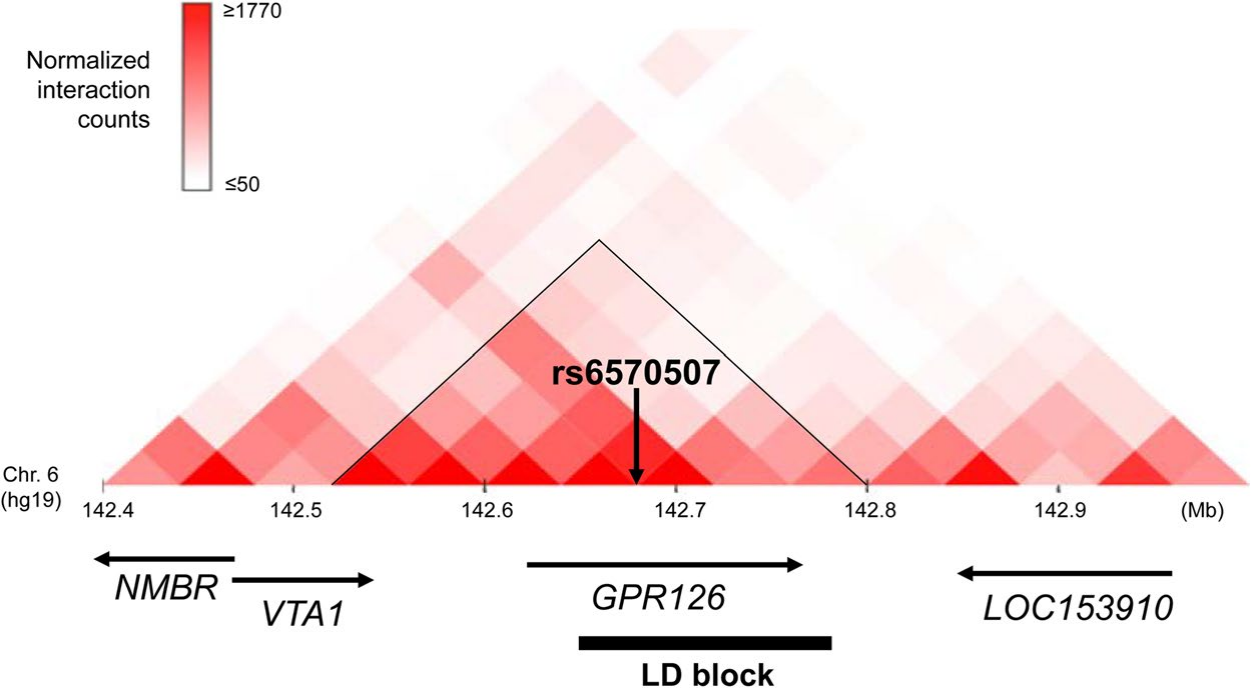
The Hi-C interaction surrounding the AIS associated region on chromosome 6q24.1. Hi-C interaction in H1-mesenchymal stem cell was generated by using the Interactive Hi-C Data Browser. *GPR126* and *VTA1* lie within the topologically associated domain (black triangle) that contains the linkage disequilibrium (LD) block (bold line) of AIS associated SNPs. An arrow indicates the location of rs6570507.

The sentinel SNP rs6570507 is present in an intron of *GPR126* (encoding G protein-coupled receptor 126), and *GPR126* is the only gene contained within the linkage disequilibrium (LD) block (*r*^2^ > 0.8) represented by rs6570507 (ref.^16^). To identify the candidate susceptibility gene in the locus, we evaluated the topologically associated domains (TADs) around the associated SNPs. Hi-C data^18^ (http://promoter.bx.psu.edu/hi-c/view.php) revealed that *GPR12*6 and *VTA1* (encoding vesicle trafficking 1) were included in the TAD that contained the LD block (Fig. 2). Using expression quantitative trait loci (eQTL) data from the Genotype-Tissue Expression (GTEx) project^19^, we investigated the target genes regulated by rs6570507. We found that the expression level of *GPR126* in subcutaneous adipose tissue and sun-exposed skin was significantly associated with rs6570507 and its risk allele rs6570507-A decreased the expression (Supplementary Fig. S2). These data strongly suggested that *GPR126* is the most plausible AIS susceptibility gene at this locus.

### Target sequencing

Recently, numerous sequencing studies of candidate genes in GWAS loci identified rare large-effect variants^20–22^. To assess the potential contribution of rare variants at the chromosome 6q24.1 locus to AIS, we carried out targeted sequencing. We screened the coding regions and exon-intron boundaries of *VTA1* (chr.6: 142468421–142539785) and *GPR126* (chr.6: 142623463–142764657) across 10,121 individuals (2,721 cases and 7,400 controls). The coverage rate of the target region, presented as an average in all individuals covered with ≥20 reads at each position, was 99.96%. We identified 183 single nucleotide variant (SNVs), 2 insertions and 9 deletions. The single-variant association analysis for each nonsynonymous variant (including missense, splice site, nonsense, and frameshift variants) with MAF <0.05 demonstrated no rare variant with significant associations (Supplementary Table S1). Subsequently, we performed the gene-based association analyses by the cohort allelic sums test (CAST)^23^ and the sequence kernel association test (SKAT)^24^, using the nonsynonymous variants. Only *GPR126* showed a nominal significant association (*P* = 4.65 × 10^−2^) with AIS by CAST; however, it did not reach the threshold for significance after Bonferroni correction **(**Supplementary Table S2).

## Discussion

In our previous study, we identified the significant association between rs6570507 and AIS in Japanese and the association is replicated in the independent cohorts of Chinese and Caucasian in the USA. However, as the numbers of Chinese and Caucasian subjects were both limited, the association between rs6570507 and AIS in these populations does not provide convincing evidence. In the current study, we have conducted a meta-analysis using eight independent multi-ethnic-cohorts and showed significant and consistent association of rs6570507 with AIS across the different ethnic groups. Recently, evidence for association of rs6570507 with AIS was reported in a Chinese cohort of Zhejiang^25^; however, we did not use the data for our meta-analysis because the allele frequency of rs6570507 in the control group of the study was quite different from the frequency for the Chinese in the public database (for example, the 1000 Genomes Project) and the minor allele was opposite. Nevertheless, our meta-analysis demonstrates convincing association between rs6570507 and AIS susceptibility.

For some common complex diseases, rare large-effect variants have recently been identified by targeted exon sequencing of genes within GWAS associated loci^26,27^. This approach is helpful to identify causal genes and to explain a further portion of disease variance. In the present study, we assessed the contribution of rare variants in the candidate genes at the locus; however, we could not find any statistically significant association in individual variants. *GPR126* showed a nominally significant association by a gene-based study. The frequency of rare and low-frequency nonsynonymous variants was increased in cases, suggesting that AIS is caused by functional impairment of the *GPR126* gene. However, in the current study, the evidence for association did not surpass Bonferroni correction. This negative result may be due to the insufficient sample size in the present study and further larger studies are required to evaluate the effect of rare and low-frequency variants in *GPR126*.

To identify the susceptibility gene at the chromosome 6q24.1 locus, we evaluated the TADs around the associated SNPs using the Hi-C database. The data showed that *GPR126* and *VTA1* were involved in the TAD that contained the LD block of the AIS associated SNPs. All SNPs strongly correlated with rs6570507 (*r*^2^ > 0.8) were present in the *GPR126* gene region. The eQTL data indicated that these SNPs were significantly associated with the expression level of *GPR126* in some tissues, such as subcutaneous adipose tissue and sun-exposed skin. It is well known that AIS patients are associated with low body weight and low body mass index (BMI)^28,29^, and are also reported to be significantly associated with lower body fat^30^. These observations suggest that GPR126 has a potential to affect AIS susceptibility through the adipose tissues. However, tissues used in GTEx project are limited, and the sample size is different by tissue. Therefore, it may fail to identify tissues contributing to the disease pathology. In order to more optimally determine the effect of the associated SNPs on scoliosis, eQTL analysis in AIS-related tissues such as intervertebral disc, cartilage and bone should be performed. Moreover, our previous experiments indicated that *GPR126* is highly expressed in the cartilage of human and proliferating chondrocytes of the vertebral body in mouse embryo^16^. Recently, Karner *et al*. reported that the mice with a conditional deletion of *Gpr126* in the cartilage displayed the clinical features of AIS including postembryonic onset of spine curvatures with rotation and absence of vertebral malformations at birth^31^. These lines of evidence strongly suggested that the *GPR126* is a susceptibility gene for AIS and its loss of function is implicated in the pathogenesis of AIS. Further studies are necessary to identify the causal variant at the locus and clarify its functional impact on the function of *GPR126*.

## Materials and Methods

### Subjects and genotyping

Informed consent was obtained from all subjects participating in this study. This study was approved by the ethics committee of the RIKEN center for Integrative Medical Sciences and all experiments were performed in accordance with relevant guidelines and regulations. All AIS subjects met clinical criteria including scoliosis with Cobb angle of 10° or more on standing spinal postero-anterior (P-A) radiographs and excluding other non-idiopathic forms of scoliosis. The subjects in the Japanese and Nanjing-Chinese cohorts were recruited and genotyped as previously described^16^. The details of additional studies: *i.e*., Guangzhou, Hong Kong, Beijing, USA, and Scandinavia studies were described as below.

### Guangzhou study

AIS subjects were recruited from the First Affiliated Hospital and Sun Yat-sen Memorial Hospital of Sun Yat-sen University. They provided detailed histories, accepted physical examinations, underwent standard up-standing P-A radiography of the whole spine, and other testing such as magnetic resonance imaging (MRI), computed tomography (CT) and nuclear scintigraphy, when necessary. All AIS subjects were diagnosed at ages 10–16 years. Control subjects were recruited from individuals who received scoliosis screening at middle and primary schools in Guangzhou and fracture patients selected from the participating hospitals. All subjects were confirmed for not having AIS by x-ray scans of the spine. Routine history and physical examinations were also conducted to exclude other relevant diseases. Genomic DNA was extracted from blood using DNA Blood Mini-kit (Tiangen Biotech, Beijing, China). Primer extension sequencing (SNaPshot) assay (Applied Biosystems, CA, USA) was used for genotyping and the results were analyzed by GeneMarker software (SoftGenetics LLC, PA, USA) at Beijing Genomics Institute (Shenzhen, China) and checked by visual inspection of I.K. and H.D.

### Hong Kong study

AIS subjects were recruited from the Duchess of Kent Children’s Hospital in Hong Kong. The inclusion criteria were as previously described^32^. Control subjects were randomly selected from the subjects recruited for the Genetic Study of Degenerative Disc Disease project^33^. All were confirmed not to have AIS by MRI examination of the spine. Genomic DNA was extracted from peripheral blood lymphocytes using standard procedures. The PCR-based invader assay (Third Wave Technologies, WI, USA) was used for genotyping.

### Beijing Study

AIS subjects were recruited from Peking Union Medical College Hospital. All subjects underwent clinical and radiologic examination such as whole spine X-ray, CT and MRI by expert spinal surgeons. Control subjects were recruited from in-house control bank. Genomic DNA was extracted from peripheral blood using the QIAamp DNA Blood Mini Kit (Qiagen, Hilden, Germany). The MassARRAY system (Sequenom; BioMiao Biological Technology, Beijing, China) was used for genotyping.

### USA Study

AIS subjects were recruited at Texas Scottish Rite Hospital for Children as previously described^10^ and genotyped using the Illumina HumanCoreExome Beadchip array. For controls, we utilized a single dataset of individuals downloaded from the dbGaP web site (http://www.ncbi.nlm.nih.gov/sites/entrez?db=gap) from Geisinger Health System-MyCode, eMERGE III Exome Chip Study under phs000957.v1.p1 (https://www.ncbi.nlm.nih.gov/projects/gap/cgi-bin/study.cgi?study_id=phs000957.v1.p1). The dbGaP controls were previously genotyped on the same microarray platform used for cases. Only subjects of self-reported Non-Hispanic White were included in the present study. Phenotypes of all controls were reviewed to exclude any with musculoskeletal or neurological disorders. We applied initial per sample quality control measures and excluded sex inconsistencies and any with missing genotype rate per person more than 0.03. Remaining samples were merged using the default mode in PLINK.1.9 (ref.^34^). Duplicated or related individuals were removed as previously described^35^. We used principal component analysis (PCA)^36^ on the merged data projected onto HapMap3 samples to correct possible stratification^37^. After quality controls, 8,647 subjects (1,360 AIS subjects and 7,287 controls) were included for the current study. We applied initial per-SNPs quality control measures using PLINK including genotyping call-rate per marker (>95%), minor allele frequency (>0.01) and deviation from Hardy-Weinberg equilibrium (cutoff p-value = 10^−4^). We noted that the SNP success rate was 99.99% for the rs6570507 and there was no significant difference in the missingness rate between cases and controls after quality controls.

### Scandinavia study

AIS subjects were recruited from six hospitals in Sweden and one in Denmark as described previously to the Scoliosis and Genetics in Scandinavia (ScoliGeneS) study^15,38–40^. Individuals with a history or clinical sign of a non-idiopathic scoliosis and with neural abnormalities in a MRI of the spine were excluded. All control subjects were females and recruited from the Osteoporosis Prospective Risk Assessment cohort and PEAK-25 cohort^41,42^. Dual-energy X-ray absorptiometry (DXA) scan was performed in both cohorts and subjects showing any sign of a curved spine on DXA were excluded. Genomic DNA was extracted from blood or saliva using the QIAamp 96 DNA Blood Kit and the Autopure LS system (Qiagen). iPLEX Gold chemistry and the MassARRAY system (Agena Bioscience, CA, USA) were used for genotyping. Genotype calls were checked by two persons individually using the MassARRAY Typer v4.0 Software (Agena Bioscience).

### Statistical analysis

The association between rs6570507 and AIS in each study was evaluated by the Cochrane-Armitage trend test. Data from the eight studies were combined using the inverse-variance method assuming a fixed-effects model in the METAL software package (http://csg.sph.umich.edu//abecasis/Metal/)^43^. The heterogeneity among studies was tested using Cochran’s Q test based upon inverse variance weights using METAL.

### Target sequencing

Cases were recruited from collaborating hospitals of Japan Scoliosis Clinical Research Group (JSCRG)^8,9^, and controls were recruited from the BioBank Japan project, the Osaka-Midosuji Rotary Club and the PharmaSNP Consortium^44,45^. Multiplex PCR-based target sequencing was carried out as previously described^45^. Primers were designed using the Primer 3 software (ver. 2.3.4) to obtain 180–300 bp PCR products. We amplified 9,847 bp consisting of 47 genomic fractions using three multiplex-PCR products with dual barcodes for each individual. After purifying of each library using Agencourt AMPure XP (Beckman Coulter, CA, USA), the library was applied to the bioanalyzer (Agilent Technologies, CA, USA) to check the size distribution and quantified using the KAPA library quantification kit (Kapa Biosystems, MA, USA) on an ABI Prism 7700 sequence detection system (Life Technologies, CA, USA). Sequencing was carried out using the HiSeq. 2500 instrument (Illumina) with 2 × 150-bp paired-end reads and dual 8-bp barcode sequences. Sequence reads were aligned to the human reference sequence (hg19) by Burrows-Wheeler Aligner (ver. 0.7.9a) and then applied to the RealignerTargetCreator and IndelRealigner tools using GATK (ver. 3.2.2) for each bam file. The quality control was performed as previously described^45^. We selected variants that clearly showed three peaks corresponding to three genotypes and two peaks if they were considered rare variants by visual inspection.

## Electronic supplementary material

Supplementary Information
