# Supplementary material for: A multi-ethnic meta-analysis confirms the association of rs6570507 with adolescent idiopathic scoliosis
Source: Sci Rep. 2018 Aug 1;8:11575. doi: 10.1038/s41598-018-29011-7 (PMC6070519; doi:10.1038/s41598-018-29011-7)
Supplement: Supplementary file 1 — Supplementary Information [file 41598_2018_29011_MOESM1_ESM.pdf]

# Supplementary Information

## A multi-ethnic meta-analysis confirms the association of rs6570507 with adolescent idiopathic scoliosis


Ikuyo Kou[1], Kota Watanabe[2,*], Yohei Takahashi[1,2], Yukihide Momozawa[3], Anas Khanshour[4], Anna Grauers[5,6], Hang Zhou[7], Gang Liu[8], Yan-Hui Fan[9], Kazuki Takeda[1,2], Yoji Ogura[1,2], Taifeng Zhou[7], Yusuke Iwasaki[3], Michiaki Kubo[3], Zhihong Wu[10,11,12], Morio Matsumoto[2], Japan Scoliosis Clinical Research Group (JSCRG)[#], Texas Scottish Rite Hospital for Children Clinical Group (TSRHCCG)[#], Elisabet Einarsdottir[13,14], Juha Kere[13,14,15], Dongsheng Huang[16], Guixing Qiu[8,11,12], Yong Qiu[17], Carol A. Wise[4,18,19,20] You-Qiang Song[9], Nan Wu[8,11,12], Peiqiang Su[7], Paul Gerdhem[6,21], Shiro Ikegawa[1,*]

[1]Laboratory of Bone and Joint Diseases, RIKEN Center for Integrative Medical Sciences, Tokyo, Japan
[2]Department of Orthopaedic Surgery, Keio University School of Medicine, Tokyo, Japan
[3]Laboratory for Genotyping Development, RIKEN Center for Integrative Medical Sciences, Yokohama, Japan
[4]Sarah M. and Charles E. Seay Center for Musculoskeletal Research, Texas Scottish Rite Hospital for Children, Dallas, Texas, USA
[5]Department of Orthopaedics, Sundsvall and Härnösand County Hospital, Sundsvall, Sweden
[6]Department of Clinical Science, Intervention and Technology (CLINTEC) Karolinska Institutet, Stockholm, Sweden
[7]Department of Spine Surgery, The First Affiliated Hospital of Sun Yat-Sen University, Guangzhou, China
[8]Department of Orthopedic Surgery, Peking Union Medical College Hospital, Peking Union Medical College and Chinese Academy of Medical Sciences, Beijing, China
[9]Department of Biochemistry, University of Hong Kong, Hong Kong, China
[10]Department of Central Laboratory, Peking Union Medical College Hospital, Peking Union Medical College and Chinese Academy of Medical Sciences, Beijing, China
[11]Beijing Key Laboratory for Genetic Research of Skeletal Deformity, China
[12]Medical Research Center of Orthopedics, Chinese Academy of Medical Sciences, Beijing, China
[13]Folkhälsan Institute of Genetics, and Molecular Neurology Research Program, University of Helsinki, Helsinki, Finland
[14]Department of Biosciences and Nutrition, Karolinska Institutet, Huddinge, Sweden
[15]Department of Medical and Molecular Genetics, King's College London, Guy's Hospital, London, UK
[16]Department of Orthopedics, Sun Yat-Sen Memorial Hospital of Sun Yat-Sen University, Guangzhou, China
[17]Department of Spine Surgery, The Affiliated Drum Tower Hospital of Nanjing University Medical School, Nanjing, China
[18]McDermott Center for Human Growth and Development, University of Texas Southwestern Medical Center at Dallas, Dallas, Texas, USA
[19]Department of Pediatrics, University of Texas Southwestern Medical Center at Dallas, Dallas, Texas, USA
[20]Department of Orthopaedic Surgery, University of Texas Southwestern Medical Center at Dallas, Dallas, Texas, USA
[21]Department of Orthopaedics, Karolinska University Hospital, Stockholm, Sweden

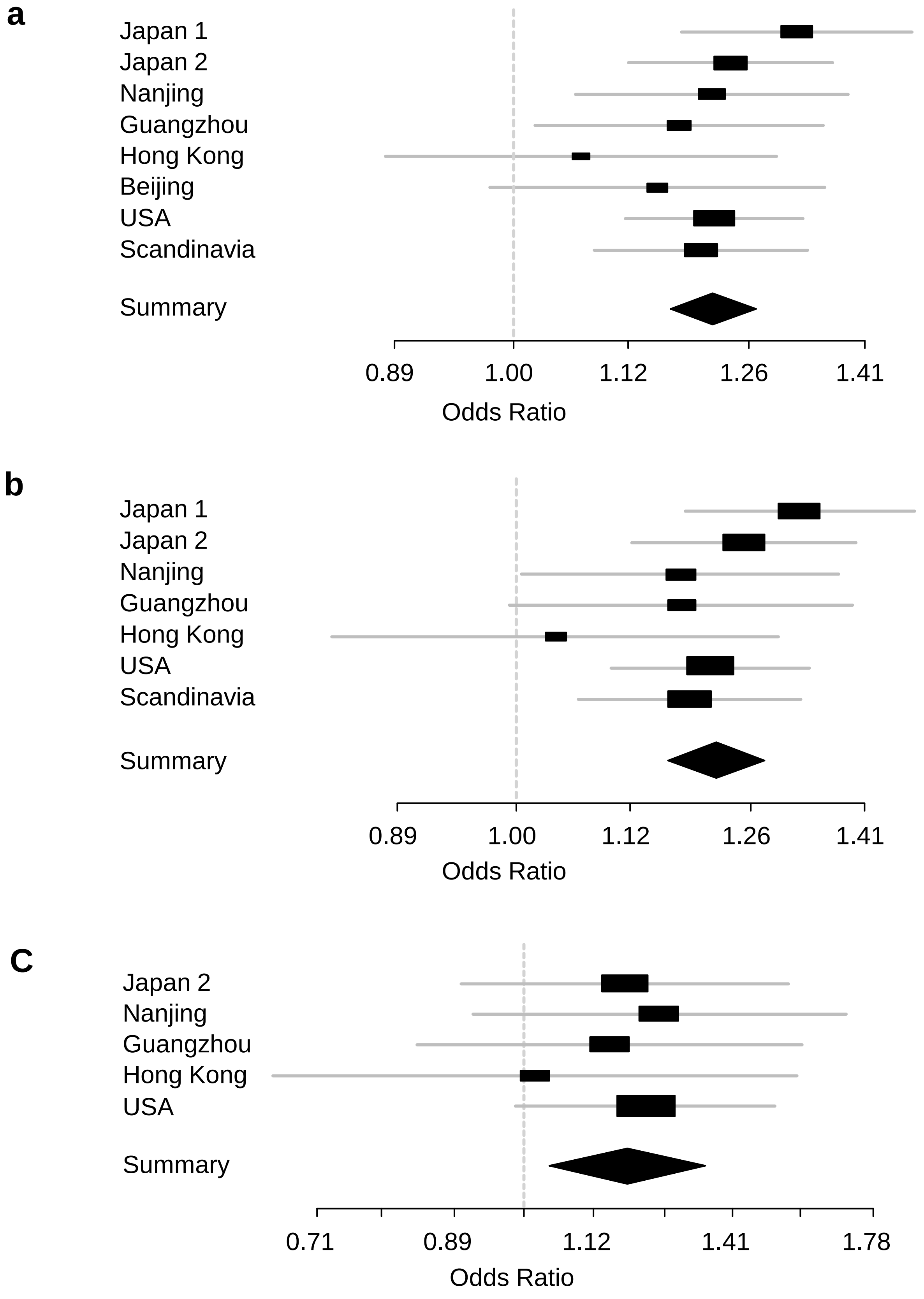


**Figure S1. Forest plots for the meta-analysis of the association between rs6570507 and AIS.** (a) all subjects, (b) female and (c) male. Odds ratios and confidence intervals were calculated based on the fixed-effect model. The contributing effect from each study is shown by a square with its confidence interval indicated by a horizontal line. Summary: the combined meta-analysis estimate.

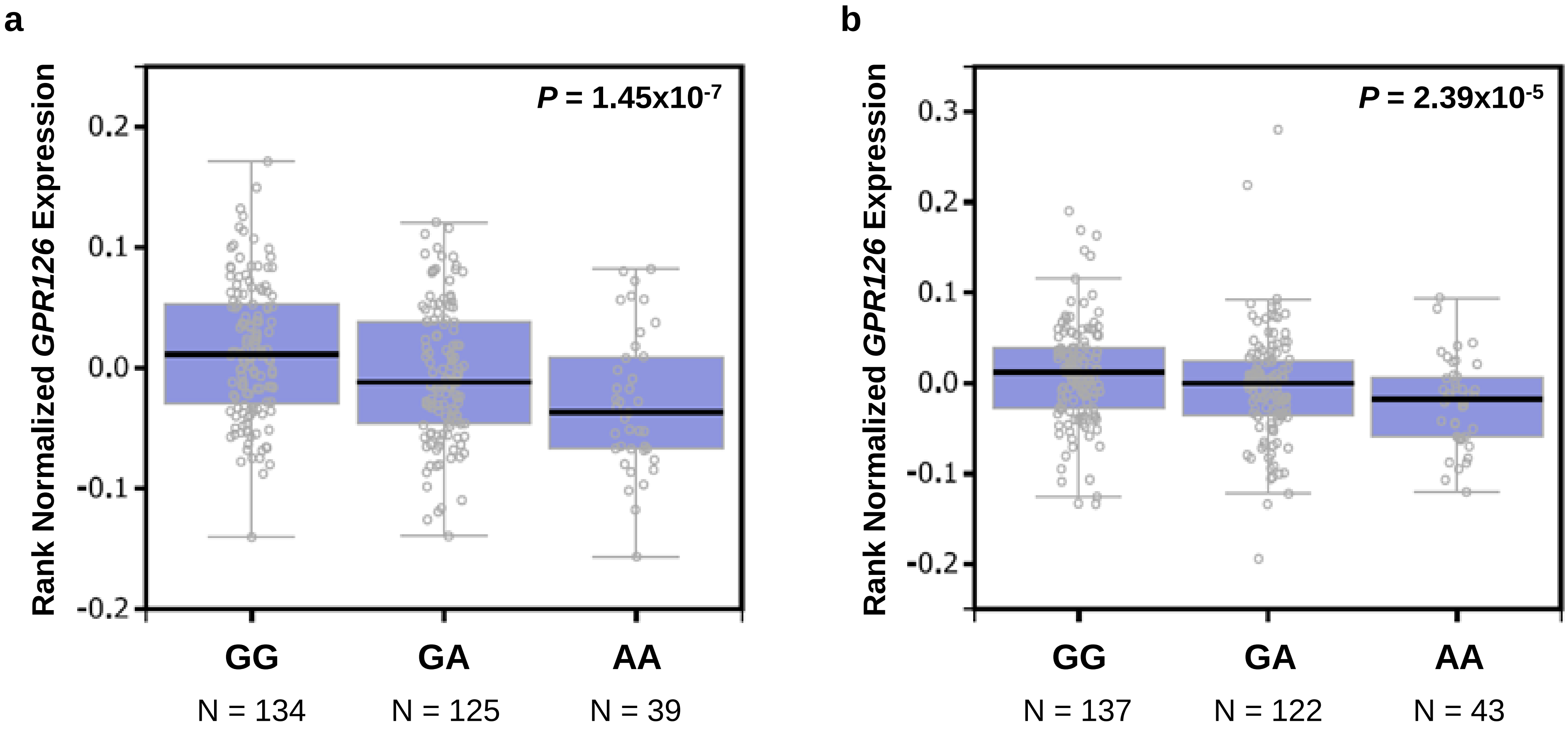


**Figure S2. Expression quantitative trait loci (eQTL) analysis for *GPR126*.**

Box plots of associations between genotypes of rs6570507 with *GPR126* expression in (a) adipose subcutaneous and (b) sun-exposed (lower leg) skin from the GTEx Portal (v6p). Expression matrices were derived from RPKM values at the gene level. Expression values were quantile normalized to the average empirical distribution observed across samples and then transformed to quantiles of standard normal distribution. Each grey circle represents an individual. The medians are indicated as black horizontal lines in the box plot.

**Table S1. Association results of nonsynonymous variants in single variant analysis.**

| Chr | Position | Ref | Alt | Variant name | Allele frequency | | *P* value | OR | Gene | Type |
|---|---|---|---|---|---|---|---|---|---|---|
| | | | | | Case | Control | | | | |
| 6 | 142,468,429 | C | G | | 0.00037 | 0.00007 | 0.178 | 5.440 | VTA1 | NON_SYNONYMOUS_CODING |
| 6 | 142,468,441 | C | A | rs781192805 | 0.00018 | 0.00000 | 0.269 | Inf | VTA1 | NON_SYNONYMOUS_CODING |
| 6 | 142,468,447 | C | G | | 0.00037 | 0.00041 | 1.000 | 0.907 | VTA1 | NON_SYNONYMOUS_CODING |
| 6 | 142,468,510 | A | G | rs531459447 | 0.00018 | 0.00014 | 1.000 | 1.360 | VTA1 | NON_SYNONYMOUS_CODING |
| 6 | 142,468,519 | C | T | | 0.00000 | 0.00007 | 1.000 | 0.000 | VTA1 | NON_SYNONYMOUS_CODING |
| 6 | 142,487,409 | A | G | | 0.00055 | 0.00074 | 0.772 | 0.742 | VTA1 | NON_SYNONYMOUS_CODING |
| 6 | 142,490,796 | G | A | rs2232302 | 0.00000 | 0.00007 | 1.000 | 0.000 | VTA1 | NON_SYNONYMOUS_CODING |
| 6 | 142,490,816 | T | C | | 0.00000 | 0.00007 | 1.000 | 0.000 | VTA1 | SPLICE_SITE_DONOR |
| 6 | 142,491,497 | C | T | | 0.00000 | 0.00007 | 1.000 | 0.000 | VTA1 | NON_SYNONYMOUS_CODING |
| 6 | 142,491,533 | C | T | | 0.00000 | 0.00014 | 1.000 | 0.000 | VTA1 | NON_SYNONYMOUS_CODING |
| 6 | 142,491,536 | T | G | | 0.00018 | 0.00000 | 0.269 | Inf | VTA1 | NON_SYNONYMOUS_CODING |
| 6 | 142,519,656 | C | A | rs557156156 | 0.00000 | 0.00007 | 1.000 | 0.000 | VTA1 | NON_SYNONYMOUS_CODING |
| 6 | 142,519,663 | A | G | rs765659030 | 0.00037 | 0.00034 | 1.000 | 1.088 | VTA1 | NON_SYNONYMOUS_CODING |
| 6 | 142,519,678 | A | G | | 0.00000 | 0.00020 | 0.569 | 0.000 | VTA1 | NON_SYNONYMOUS_CODING |
| 6 | 142,525,140 | T | C | | 0.00000 | 0.00007 | 1.000 | 0.000 | VTA1 | NON_SYNONYMOUS_CODING |
| 6 | 142,525,178 | G | A | rs143729861 | 0.00018 | 0.00000 | 0.269 | Inf | VTA1 | NON_SYNONYMOUS_CODING |
| 6 | 142,525,187 | A | G | rs199631870 | 0.00000 | 0.00007 | 1.000 | 0.000 | VTA1 | NON_SYNONYMOUS_CODING |
| 6 | 142,539,698 | G | A | | 0.00000 | 0.00007 | 1.000 | 0.000 | VTA1 | NON_SYNONYMOUS_CODING |
| 6 | 142,539,758 | TACTG | T | | 0.00018 | 0.00000 | 0.269 | Inf | VTA1 | FRAME_SHIFT |
| 6 | 142,630,694 | G | A | | 0.00018 | 0.00000 | 0.269 | Inf | GPR126 | NON_SYNONYMOUS_CODING |
| 6 | 142,630,698 | G | A | | 0.00000 | 0.00007 | 1.000 | 0.000 | GPR126 | NON_SYNONYMOUS_CODING |
| 6 | 142,630,707 | G | A | | 0.00018 | 0.00000 | 0.269 | Inf | GPR126 | NON_SYNONYMOUS_CODING |
| 6 | 142,630,712 | C | T | | 0.00000 | 0.00007 | 1.000 | 0.000 | GPR126 | NON_SYNONYMOUS_CODING |
| 6 | 142,688,969 | A | G | rs17280293 | 0.04006 | 0.03500 | 0.090 | 1.150 | GPR126 | NON_SYNONYMOUS_CODING |
| 6 | 142,689,030 | A | T | | 0.00000 | 0.00007 | 1.000 | 0.000 | GPR126 | NON_SYNONYMOUS_CODING |
| 6 | 142,691,312 | G | A | rs778631441 | 0.00000 | 0.00020 | 0.569 | 0.000 | GPR126 | NON_SYNONYMOUS_CODING |
| 6 | 142,691,326 | T | A | | 0.00000 | 0.00007 | 1.000 | 0.000 | GPR126 | NON_SYNONYMOUS_CODING |
| 6 | 142,691,484 | C | T | | 0.00018 | 0.00000 | 0.269 | Inf | GPR126 | NON_SYNONYMOUS_CODING |
| 6 | 142,691,645 | G | T | | 0.00000 | 0.00007 | 1.000 | 0.000 | GPR126 | NON_SYNONYMOUS_CODING |
| 6 | 142,691,702 | A | G | rs763469625 | 0.00000 | 0.00007 | 1.000 | 0.000 | GPR126 | NON_SYNONYMOUS_CODING |
| 6 | 142,691,741 | G | A | | 0.00000 | 0.00007 | 1.000 | 0.000 | GPR126 | NON_SYNONYMOUS_CODING |
| 6 | 142,691,765 | T | A | rs747347676 | 0.00129 | 0.00074 | 0.287 | 1.732 | GPR126 | NON_SYNONYMOUS_CODING |
| 6 | 142,691,861 | G | A | rs145304917 | 0.00092 | 0.00095 | 1.000 | 0.974 | GPR126 | NON_SYNONYMOUS_CODING |
| 6 | 142,691,864 | G | A | rs373599767 | 0.00000 | 0.00007 | 1.000 | 0.000 | GPR126 | NON_SYNONYMOUS_CODING |
| 6 | 142,691,873 | A | C | | 0.00055 | 0.00007 | 0.062 | 8.184 | GPR126 | NON_SYNONYMOUS_CODING |
| 6 | 142,691,874 | A | T | rs200437948 | 0.00147 | 0.00176 | 0.847 | 0.839 | GPR126 | NON_SYNONYMOUS_CODING |
| 6 | 142,703,088 | G | T | rs544627788 | 0.00018 | 0.00000 | 0.269 | Inf | GPR126 | NON_SYNONYMOUS_CODING |
| 6 | 142,703,103 | C | T | rs184591267 | 0.00000 | 0.00007 | 1.000 | 0.000 | GPR126 | NON_SYNONYMOUS_CODING |
| 6 | 142,704,927 | C | G | | 0.00018 | 0.00007 | 0.465 | 2.720 | GPR126 | NON_SYNONYMOUS_CODING |
| 6 | 142,704,978 | A | C | | 0.00018 | 0.00014 | 1.000 | 1.360 | GPR126 | NON_SYNONYMOUS_CODING |
| 6 | 142,714,083 | A | G | | 0.00000 | 0.00007 | 1.000 | 0.000 | GPR126 | SPLICE_SITE_ACCEPTOR |
| 6 | 142,715,032 | G | A | | 0.00000 | 0.00007 | 1.000 | 0.000 | GPR126 | SPLICE_SITE_ACCEPTOR |
| 6 | 142,721,633 | G | T | rs774788882 | 0.00037 | 0.00081 | 0.378 | 0.453 | GPR126 | NON_SYNONYMOUS_CODING |
| 6 | 142,721,648 | A | C | | 0.00000 | 0.00007 | 1.000 | 0.000 | GPR126 | NON_SYNONYMOUS_CODING |
| 6 | 142,721,652 | G | T | | 0.00000 | 0.00007 | 1.000 | 0.000 | GPR126 | NON_SYNONYMOUS_CODING |
| 6 | 142,721,674 | A | T | rs139306565 | 0.00551 | 0.00527 | 0.828 | 1.046 | GPR126 | NON_SYNONYMOUS_CODING |
| 6 | 142,721,675 | C | T | | 0.00018 | 0.00000 | 0.269 | Inf | GPR126 | NON_SYNONYMOUS_CODING |
| 6 | 142,723,192 | TTAAG | T | | 0.00018 | 0.00000 | 0.269 | Inf | GPR126 | SPLICE_SITE_DONOR; STOP_GAINED |
| 6 | 142,723,777 | A | G | | 0.00000 | 0.00007 | 1.000 | 0.000 | GPR126 | NON_SYNONYMOUS_CODING |
| 6 | 142,723,807 | C | A | | 0.00000 | 0.00007 | 1.000 | 0.000 | GPR126 | NON_SYNONYMOUS_CODING |
| 6 | 142,723,886 | T | C | rs755473615 | 0.00000 | 0.00014 | 1.000 | 0.000 | GPR126 | NON_SYNONYMOUS_CODING |
| 6 | 142,723,900 | A | G | rs748484928 | 0.00092 | 0.00081 | 0.788 | 1.133 | GPR126 | NON_SYNONYMOUS_CODING |
| 6 | 142,723,918 | TC | T | | 0.00018 | 0.00000 | 0.269 | Inf | GPR126 | FRAME_SHIFT |
| 6 | 142,724,941 | C | T | | 0.00000 | 0.00007 | 1.000 | 0.000 | GPR126 | NON_SYNONYMOUS_CODING |
| 6 | 142,725,007 | C | G | rs749938643 | 0.00000 | 0.00007 | 1.000 | 0.000 | GPR126 | NON_SYNONYMOUS_CODING |
| 6 | 142,725,013 | G | A | rs753293070 | 0.00018 | 0.00000 | 0.269 | Inf | GPR126 | NON_SYNONYMOUS_CODING |
| 6 | 142,726,870 | T | A | | 0.00018 | 0.00020 | 1.000 | 0.907 | GPR126 | NON_SYNONYMOUS_CODING |
| 6 | 142,726,933 | T | C | | 0.00000 | 0.00007 | 1.000 | 0.000 | GPR126 | NON_SYNONYMOUS_CODING |
| 6 | 142,732,448 | T | C | | 0.00018 | 0.00000 | 0.269 | Inf | GPR126 | NON_SYNONYMOUS_CODING |
| 6 | 142,732,463 | C | T | | 0.00018 | 0.00000 | 0.269 | Inf | GPR126 | NON_SYNONYMOUS_CODING |
| 6 | 142,736,121 | G | A | | 0.00000 | 0.00007 | 1.000 | 0.000 | GPR126 | NON_SYNONYMOUS_CODING |
| 6 | 142,740,981 | T | C | rs371643501 | 0.00000 | 0.00007 | 1.000 | 0.000 | GPR126 | NON_SYNONYMOUS_CODING |
| 6 | 142,741,037 | A | G | rs188834899 | 0.00129 | 0.00115 | 0.819 | 1.120 | GPR126 | NON_SYNONYMOUS_CODING |
| 6 | 142,741,089 | A | G | rs775625580 | 0.00018 | 0.00000 | 0.269 | Inf | GPR126 | NON_SYNONYMOUS_CODING |
| 6 | 142,741,092 | G | A | rs536714306 | 0.00294 | 0.00196 | 0.237 | 1.502 | GPR126 | NON_SYNONYMOUS_CODING |
| 6 | 142,741,143 | C | A | rs746146861 | 0.00037 | 0.00034 | 1.000 | 1.088 | GPR126 | NON_SYNONYMOUS_CODING |
| 6 | 142,758,612 | G | A | | 0.00000 | 0.00007 | 1.000 | 0.000 | GPR126 | NON_SYNONYMOUS_CODING |
| 6 | 142,758,665 | TAACTC | T | | 0.00000 | 0.00007 | 1.000 | 0.000 | GPR126 | FRAME_SHIFT |
| 6 | 142,759,367 | C | T | | 0.00018 | 0.00000 | 0.269 | Inf | GPR126 | NON_SYNONYMOUS_CODING |
| 6 | 142,759,370 | A | G | rs770715586 | 0.00000 | 0.00020 | 0.569 | 0.000 | GPR126 | NON_SYNONYMOUS_CODING |
| 6 | 142,759,393 | A | T | | 0.00018 | 0.00000 | 0.269 | Inf | GPR126 | NON_SYNONYMOUS_CODING |
| 6 | 142,759,406 | C | A | | 0.00000 | 0.00007 | 1.000 | 0.000 | GPR126 | NON_SYNONYMOUS_CODING |
| 6 | 142,759,420 | T | C | | 0.00000 | 0.00007 | 1.000 | 0.000 | GPR126 | NON_SYNONYMOUS_CODING |
| 6 | 142,762,078 | G | A | | 0.00000 | 0.00007 | 1.000 | 0.000 | GPR126 | NON_SYNONYMOUS_CODING |
| 6 | 142,762,081 | G | A | | 0.00000 | 0.00020 | 0.569 | 0.000 | GPR126 | NON_SYNONYMOUS_CODING |
| 6 | 142,764,474 | A | G | | 0.00018 | 0.00000 | 0.269 | Inf | GPR126 | NON_SYNONYMOUS_CODING |
| 6 | 142,764,640 | C | G | | 0.00000 | 0.00014 | 1.000 | 0.000 | GPR126 | NON_SYNONYMOUS_CODING |

Variant name was assigned according to dbSNP (build 147).

Chr, chromosome; Ref, reference allele; Alt, alternative allele; OR, odds ratio.

**Table S2. The gene-based association test for nonsynonymous variants (MAF < 0.05) in *GPR126* and *VTA1*.**

| Gene | Number of variants | Carrier frequency (%) | | CAST | | SKAT |
|---|---|---|---|---|---|---|
| | | Case | Control | *P* value | OR (95% CI) | *P* value |
| GPR126 | 58 | 11.32 | 9.95 | 0.047 | 1.16 (1.00-1.33) | 0.239 |
| VTA1 | 19 | 0.51 | 0.51 | 1.000 | 1.00 (0.54-1.85) | 0.828 |

CAST, the cohort allelic sums test; SKAT, the sequence kernel association test; OR, odds ratio; CI, confidence interval.
